# Active Surveillance, Drug Resistance, and Genotypic Profiling of *Staphylococcus aureus* Among School-Age Children in China

**DOI:** 10.3389/fmed.2021.701494

**Published:** 2021-08-10

**Authors:** Bingshao Liang, Xiaoyun Liang, Fei Gao, Yan Long, Jialiang Mai, Xiaolan Ai, Jielin Wang, Xiurong Gao, Zhile Xiong, Zhuwei Liang, Chao Zhang, Sitang Gong, Zhenwen Zhou

**Affiliations:** ^1^Clinical Laboratory, Guangzhou Women and Children's Medical Center, Guangzhou Medical University, Guangzhou, China; ^2^Department of Gastroenterology, Guangzhou Women and Children's Medical Center, Guangzhou Medical University, Guangzhou, China; ^3^Department of Laboratory Medicine, Guangzhou Eighth People's Hospital, Guangzhou Medical University, Guangzhou, China

**Keywords:** antibiotic resistance, nasal colonization, methicillin-resistant *Staphylococcus aureus*, molecular epidemiology, oxacillin-susceptible MRSA, *Staphylococcus aureus*

## Abstract

Methicillin-susceptible (MSSA) and methicillin-resistant *Staphylococcus aureus* (MRSA) nasal colonization predisposes individuals for endogenous infections and is a major threat to children. Recently, oxacillin/cefoxitin-susceptible *mecA*-positive *S. aureus* (OS-MRSA) has been reported worldwide. Herein, a prospective, cross-sectional study was conducted across five schools, representing three educational stages, in Guangzhou, China. Nasal swabs from 2,375 students were cultured for *S. aureus* and all isolates were subjected to antibiotic susceptibility testing phenotypically and confirmed by *femB* and *mecA* genetic detection; all the isolates were classified as MSSA, MRSA, or OS-MRSA. All strains were also analyzed by multi-locus sequence typing. Among the 2,375 swabs, *S. aureus* was detected in 744 children (31.3%, 95% CI: 25.9–36.7%), of whom 72 had MRSA (3.0%, 95% CI: 0.6–5.4%) and 4 had OS-MRSA (0.2%, 95% CI: 0.1–0.3%), of which an oxacillin- and cefoxitin-susceptible MRSA strain was identified. The prevalence of *S. aureus* and MRSA was higher in younger children. The highest percentage of drug resistance of the *S. aureus* isolates (*n* = 744) was to penicillin (85.5%), followed by erythromycin (43.3%) and clidamycin (41.0%). The most prevalent sequence types (STs) were ST30, ST45, and ST188 in MSSA, accounting for 38.7% of the total isolates, whereas ST45, ST59, and ST338 accounted for 74.6% of the MRSA isolates and ST338 accounted for 50.0% of the OS-MRSA isolates. The MRSA and OS-MRSA isolates (*n* = 76) were grouped into three clades and one singleton, with clonal complex (CC) 45 as the most predominant linkage. The top nine multi-locus sequence typing-based CCs (CC30, CC45, CC5, CC1, CC15, CC944, CC398, CC59, CC7) represented 86.7% of all *S. aureus* isolates. All CC30 isolates were resistant to erythromycin and clidamycin, and almost all these isolates were also resistant to penicillin (99.2%). The CC45 and CC59 isolates exhibited high resistance rates to oxacillin at 31.5 and 59.0%, respectively. This study provides updated data valuable for designing effective control strategies to mitigate the burden of disease and to improve the adequacy of empirical antimicrobial treatments for potentially harmful infections.

## Introduction

Community-acquired pneumonia caused by prevalent respiratory pathogens such as *Staphylococcus aureus* is a major public health concern and leading cause of death (1, 2). Moreover, the prevalence of multidrug-resistant isolates, particularly methicillin-resistant *S. aureus* (MRSA), remains persistently high in China and is recognized as a major cause of nosocomial infections (3). The so-called “Search and Destroy” policy implemented in several countries has maintained MRSA at low endemic levels over the years (4). The anterior nares are a primary niche for *S. aureus*, and asymptomatic nasal colonization by this pathogen is a first and essential step toward the development of local and systemic disease as well as facilitates host-to-host transmission (5–7). Thus, studies aimed at determining the true prevalence of *S. aureus* nasal colonization among children in China are urgently needed.

*Staphylococcus aureus* resistant to antimicrobial drugs, particularly those that are multidrug-resistant, represent a major global concern (8) and cause a substantial burden in health care settings (9). Methicillin, the first semisynthetic penicillinase-resistant penicillin, was widely used initially until MRSA was found in England in 1961 soon after methicillin was introduced into clinical practice (10). Although methicillin was replaced by oxacillin because of its lower human toxicity and stability, the term methicillin-resistant *S. aureus* continues to be used (11). MRSA acquired drug resistance via incorporation of *mecA* or its ortholog into the chromosome at a specific site, encoding an alternative penicillin-binding protein that has low affinity for almost the entire class of β-lactam drugs including methicillin, oxacillin, and most cephem agents (12). Thus, MRSA is defined as *S. aureus* isolates genetically containing *mecA* or *mecC* or phenotypically showing resistance to oxacillin conventionally or to cefoxitin (13). Cefoxitin is a more potent inducer of *mecA* and disk diffusion tests using cefoxitin reveal clearer endpoints that are easier to read than tests with oxacillin (14). However, some clinical isolates are *mecA*-positive and oxacillin-susceptible and defined as oxacillin-susceptible MRSA (OS-MRSA) (15). Particularly, oxacillin- and cefoxitin-susceptible- *mecA*-positive *S. aureus* isolates, also known as “stealth” MRSA, have been increasingly reported worldwide; these bacteria can be easily misinterpreted as methicillin-susceptible *S. aureus* (MSSA) and convert to oxacillin-resistant when exposed to β-lactam antibiotics (16).

The prevalent clones among MRSA and MSSA vary in term of multi-locus sequencing typing (MLST); for example, ST188 was mostly found in MSSA (17), ST59 and ST8 were two major MRSA clones (18, 19). However, some clones share the same STs. For instance, ST30 was a major MRSA clone in nine Latin American countries and a MSSA clone in China at the same time (20, 21). Moreover, in China, the environmental origins of dominant lineages differed. Recently, ST5 and ST239 were found to be major lineages in hospital settings, whereas ST59 was mainly found in community settings (22). However, it was reported that the ST45 lineage has recently emerged in the community acquiring multiple antimicrobial resistance determinants (23, 24). Thus, screening for MRSA in nares may be a powerful tool for antimicrobial therapy (25) and for optimizing antibiotic usage by determining the prevalent clones and their drug resistance patterns.

This study was conducted to evaluate the prevalence, antimicrobial susceptibility pattern, and genotypic characterization of *S. aureus* populations among school-age children across five schools in Guangzhou, the largest city in southern China. These findings may help in designing control strategies and improving the adequacy of empirical antimicrobial treatments of potentially harmful infections.

## Materials and Methods

### Study Design

A total of 2,375 elementary and middle school-aged students (6–18 years) was recruited at five schools in < city>Guangzhou < /city>, China, in 2018 and enrolled in this prospective, cross-sectional study. None of the volunteers displayed signs of respiratory infection. Guangzhou has a high population density, along with high temperature and humidity that is conducive for *S. aureus* nasal colonization. Sterile flocked swabs moistened with sterile normal saline were inserted into both anterior nares of each participant and the nasal mucus specimen was collected by gentle rotation three times. The samples were delivered to the laboratory within 2 h of collection and processed immediately after arrival. The Ethics Committee of the Guangzhou Women and Children's Medical Center approved the study protocol (registration no. 2016081029). All participants were recruited voluntarily and provided written informed consent.

### Isolation and Identification of Bacteria

The nasal swabs were inoculated onto sheep blood agar and incubated at 35°C with 5% CO_2_ for 48 h. Based on the morphology and coagulase test results, the suspected colonies were sub-cultured on Columbia Blood Agar Medium. Next, the colonies were further identified for the presence of *S. aureus* by matrix-assisted laser desorption-time-of-flight mass spectrometry identification with a VITEK MS system (bioMérieux, Marcy l'Étoile, France). The bacterial genomic DNA of all isolates was extracted as previously described (24). All isolates were further confirmed by detection of *femB* and *mecA* by polymerase chain reaction using primers designed by Jonas et al. (26).

### Antibiotic Susceptibility Tests

All *S. aureus* isolates were tested for susceptibility to 16 antibiotics using GP67 cards by the automated VITEK2 compact system (bioMérieux) as previously reported (24), including penicillin (PEN), oxacillin (OXA), erythromycin (ERY), clindamycin (CLI), sulfamethoxazole-trimethoprim (SXT), gentamicin (GEN), vancomycin (VAN), ciprofloxacin (CIP), levofloxacin (LVX), moxifloxacin (MFX), tetracycline (TCY), nitrofurantoin (NIT), rifampicin (RIF), tigecycline (TGC), linezolid (LZD), and quinupristin/dalfopristin (QDA). The minimum inhibitory concentrations of the antimicrobial agents were obtained and interpretative criteria (breakpoints) for these agents were according to the Clinical and Laboratory Standards Institute guidelines (13). All isolates were classified as MSSA, MRSA, or OS-MRSA by oxacillin/cefoxitin susceptibility tests and detection of *mecA* simultaneously. *Staphylococcus aureus* ATCC 29213 was used for quality control. All isolates were tested for inducible clindamycin resistance (D test). Multidrug-resistant *S. aureus* was defined as a strain that was non-susceptible to ≥1 drug in at least three antimicrobial categories (27).

### MLST and Data Analysis

MLST genotyping of all isolates was performed as previously described (24). Each isolate was assigned an ST number, and novel sequence types were assigned and included in the MLST database at https://pubmlst.org/saureus/. The STs were grouped into clonal complexes (CCs) based on the designation published at https://pubmlst.org/organisms/staphylococcus-aureus/clonal-complexes. The associated STs shared by six of the seven loci were included into the same CCs (14). CC59, CC7, CC25, CC121, and CC398 were assigned according to previous publications (22, 28, 29). CC944 was assigned in this study because its associated ST, ST944, was the first reported type to the best of our knowledge. All isolates, including 72 MRSA, 4 OS-MRSA, and 668 MSSA isolates, were clustered based on the MLST data using the minimum spanning tree method in BioNumerics software (Applied Maths, Sint-Martens-Latem, Belgium). The unweighted pair group method with arithmetic mean was applied to design a dendrogram and antibiogram of the 72 MRSA and 4 OS-MRSA isolates using BioNumerics software. All the work regarding the handling of *S. aureus* was performed in a biosafety level 2 laboratory at Guangzhou Women and Children's Medical Center, and the person who performed the experiments had specific training and the biosafety level 2 standard microbiological regulations were strictly followed.

### Statistical Analysis

The Chi-squared test or Fisher's exact test was applied to compare the categorical variables using SPSS 17.0 software (SPSS, Inc., Chicago, IL, USA). *P* ≤ 0.05 indicated a statistically significant difference.

## Results

### Nasal Carriage Rates of *S. aureus*, MRSA, and OS-MRSA Among Children in Guangzhou, China

From October to November 2018, 2,375 eligible participants were enrolled across five schools in Guangzhou, southern China, including three elementary schools (*n* = 1,029), one junior middle school (*n* = 592), and one senior middle school (*n* = 754). Table 1 shows the nasal carriage rates among the participating students. The prevalence of *S. aureus* and MRSA nasal carriage was high among students across all educational stages with an average of 31.3% [95% confidence interval (CI): 25.9–36.7%] and 3.0% (95% CI: 0.6–5.4%), respectively, whereas OS-MRSA was relatively low at 0.2% (95% CI: 0.1–0.3%). The carriage rates of *S. aureus* and MRSA significantly differed among the three educational stages (*P* < 0.01). Moreover, the prevalence of *S. aureus* and MRSA was dependent on age, with significantly higher rates among students in earlier educational stages (Table 1).

**Table 1:** Nasal carriage rates of *Staphylococcus aureus*, MRSA, and OS-MRSA among students aged 6–18 years.

**Educational stage**	**Students screened (*N*)**	***S. aureus*^**a**^ ratio**	**MRSA^**b**^ ratio**	**OS-MRSA ratio**
Elementary school	1,029	35.3	4.2	0.3
Junior middle school	592	30.9	3.2	0.0
Senior middle school	754	26.3	1.3	0.1
Total	2,375	31.3	3.0	0.2

*Different lowercase letters (a, b) indicate a significance difference (P < 0.01) of S. aureus, MRSA prevalence among children from three educational stages, determined with the Chi-squared test*.

*MRSA, methicillin-resistant S. aureus; OS-MRSA, cefoxitin/oxacillin-susceptible MRSA*.

### Antimicrobial Susceptibility Testing

The highest percentage of drug resistance of *S. aureus* isolates (*n* = 744) was penicillin (PEN, 85.5%), followed by erythromycin (ERY, 43.3%), clindamycin (CLI, 41.0%), tetracycline (TCY, 8.7%), sulfamethoxazole-trimethoprim (SXT, 3.5%), rifampicin (RIF, 1.6%), ciprofloxacin (CIP, 0.9%), levofloxacin (LVX, 0.9%), moxifloxacin (MFX, 0.7%), gentamicin (GEN, 0.7%), and linezolid (LZD, 0.1%). None of the isolates was resistant to vancomycin, nitrofurantoin, tigecycline, or quinupristin/dalfopristin. Compared with the MSSA group, the resistant rates to penicillin, erythromycin, clindamycin, tetracycline, and rifampicin in the MRSA group were significantly higher (Table 2). The overall multidrug-resistance rate in this study was 13.6%.

**Table 2:** Antibiotic susceptibility tests for 744 *Staphylococcus aureus* isolates from students aged 6–18 years.

**Antibiotic**	***S. aureus*** **(** ***N*** **=** **744)**		**MRSA/OS-MRSA (** ***N*** **=** **76)**		**MSSA (** ***N*** **=** **668)**		***P*-value**
	**R, *N* (%)**	**I, *N* (%)**	**R, *N* (%)**	**I, *N* (%)**	**R, *N* (%)**	**I, *N* (%)**	
Penicillin	636 (85.5)	0 (0.0)	75 (98.7)	0 (0.0)	561 (84.0)	0 (0.0)	<0.01^a^
Erythromycin	322 (43.3)	0 (0.0)	48 (63.2)	0 (0.0)	274 (41.0)	0 (0.0)	<0.01^a^
Clindamycin	305 (41.0)	0 (0.0)	45 (59.2)	0 (0.0)	260 (38.9)	0 (0.0)	<0.01^a^
SXT	26 (3.5)	0 (0.0)	2 (2.6)	0 (0.0)	24 (3.6)	0 (0.0)	>0.05^b^
Gentamicin	5 (0.7)	8 (1.1)	1 (1.3)	1 (1.3)	4 (0.6)	7 (1.0)	>0.05^b^
Vancomycin	0 (0.0)	0 (0.0)	0 (0.0)	0 (0.0)	0 (0.0)	0 (0.0)	NA
Ciprofloxacin	7 (0.9)	6 (0.8)	2 (2.6)	2 (2.6)	5 (0.7)	4 (0.6)	<0.05^b^
Levofloxacin	7 (0.9)	0 (0.0)	2 (2.6)	0 (0.0)	5 (0.7)	0 (0.0)	>0.05^c^
Moxifloxacin	5 (0.7)	3 (0.4)	2 (2.6)	0 (0.0)	3 (0.4)	3 (0.4)	>0.05^c^
Tetracycline	65 (8.7)	1 (0.1)	13 (17.1)	1 (1.3)	52 (7.8)	0 (0.0)	<0.01^a^
Nitrofurantoin	0 (0.0)	1 (0.1)	0 (0.0)	0 (0.0)	0 (0.0)	1 (0.1)	NA
Rifampicin	12 (1.6)	87 (11.7)	2 (2.6)	33 (43.4)	10 (1.5)	54 (8.1)	<0.01^a^
Tigecycline	0 (0.0)	0 (0.0)	0 (0.0)	0 (0.0)	0 (0.0)	0 (0.0)	NA
Linezolid	1 (0.1)	0 (0.0)	0 (0.0)	0 (0.0)	1 (0.1)	0 (0.0)	NA
QDA	0 (0.0)	0 (0.0)	0 (0.0)	0 (0.0)	0 (0.0)	0 (0.0)	NA

*a, b, and c indicate antibiotics susceptibility of MRSA and OS-MRSA vs. MSSA by Chi-square test (two-sided), by continuity correction (two-sided), and by Fisher's exact test (two-sided), respectively*.

*I, intermediate resistant; R, resistant. MRSA, methicillin-resistant S. aureus; MRSA, methicillin-sensitive S. aureus; NA, not applicable; OS-MRSA, cefoxitin/oxacillin-susceptible MRSA. SXT, sulfamethoxazole-trimethoprim, QDA, quinupristin/dalfopristin*.

### MLST and CC of All *S. aureus* Isolates

**Figure 1:**
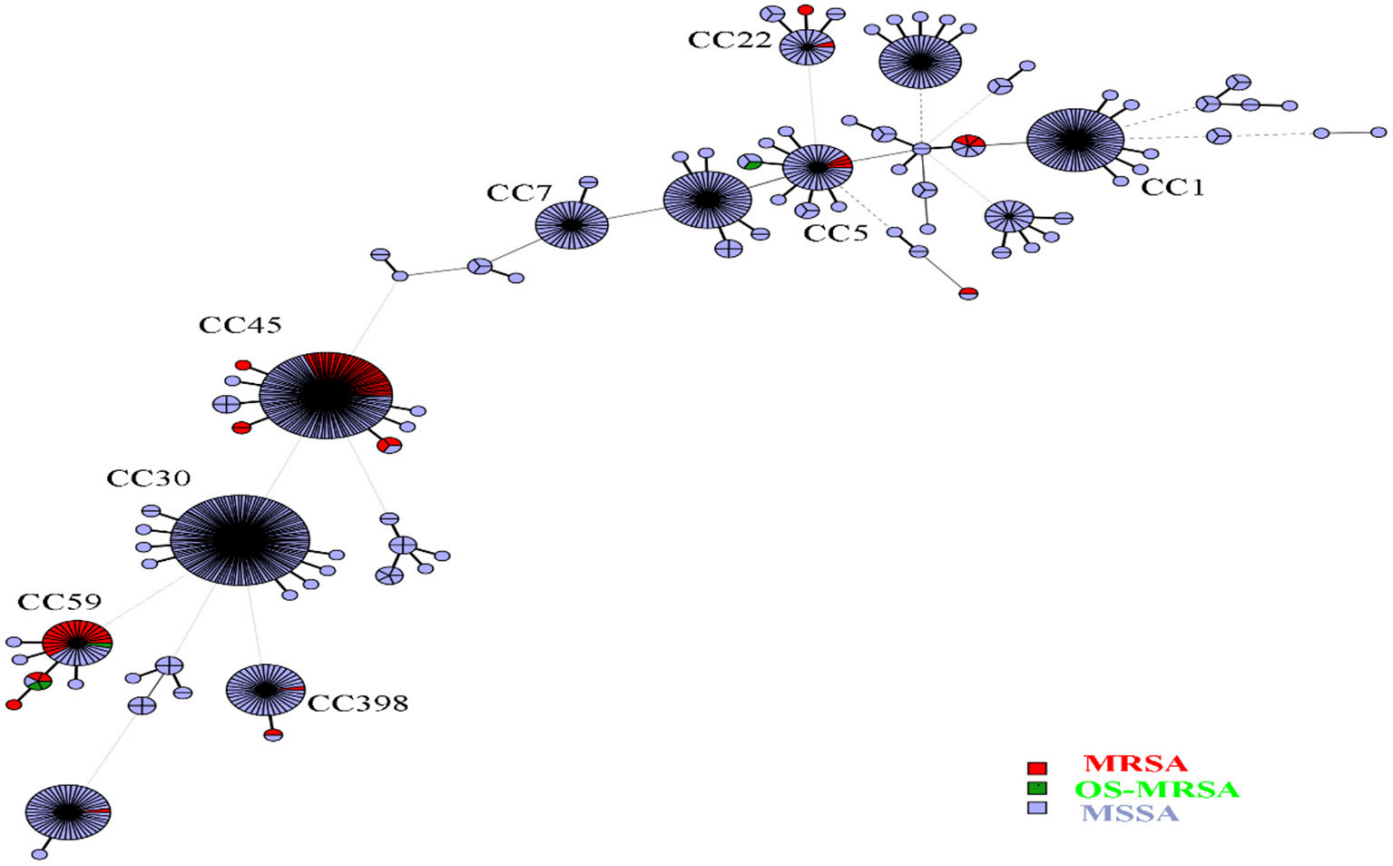
Minimum spanning tree based on the genotypic structure of all *Staphylococcus aureus* isolates. Each sequence type is represented by a node. The size of each node represents the number of *S. aureus* strains with this genotype. The length of two nodes indicates the genetic distance between the two genotypes. Labeled nodes are the top nine clonal complexes. Red, green, and blue circles represent clonal complexes that were methicillin-resistant (MRSA), cefoxitin/oxacillin-susceptible *mecA*-positive (OS-MRSA), and methicillin-susceptible *S. aureus* (MSSA), respectively.

**Figure 2:**
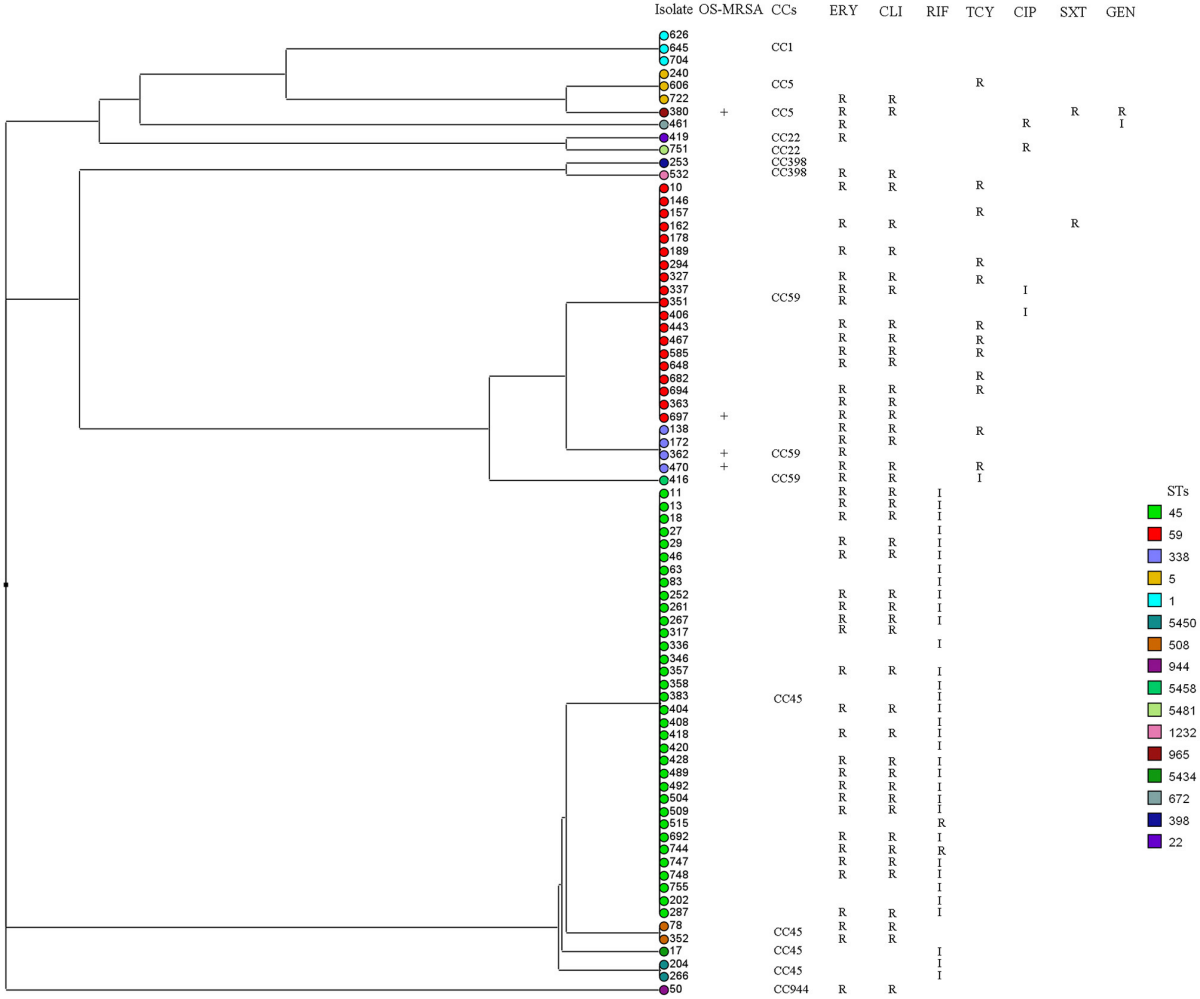
Dendrogram and antibiogram of 72 methicillin-resistant (MRSA) and four cefoxitin/oxacillin-susceptible *mecA*-positive (OS-MRSA) *Staphylococcus aureus* isolates were generated based on multi-locus sequence typing data. All isolates (*n* = 76) were grouped into three clades and one singleton. I, intermediate resistant; R, resistant. CIP, ciprofloxacin; CLI, clindamycin; ERY, erythromycin; GEN, gentamicin; RIF, rifampicin; SXT, sulfamethoxazole-trimethoprim; TCY, tetracycline.

Overall, 91 sequence types (STs) of *S. aureus* were identified, including 37 novel STs. ST30, ST45, and ST188 were the top three STs identified, representing 39.4% of all isolates. Among the MSSA isolates, ST30, ST45, and ST188 were the most prevalent, accounting for 38.7%; in turn, ST45, ST59, and ST338 accounted for 74.6% of MRSA isolates, and ST338 accounted for 50.0% of OS-MRSA isolates. In this study, 37 novel STs (ST5434 to ST5468 and ST5473, ST5481) were assigned. Many novel STs were single-locus variants of ST30, ST1, ST45, ST59, ST22, and ST15, belonging to CC30, CC1, CC45, CC59, CC22, and CC15, respectively. The 37 newly assigned STs were distributed in 49 strains, including five MRSA and 44 MSSA. Three MRSA isolates belonged to CC45, the other two belonged to CC22 and CC59. All *S. aureus* isolates could be assigned to 16 CCs; however, the top nine CCs (CC30, CC45, CC5, CC1, CC15, CC944, CC398, CC59, and CC7) represented 86.7% of all isolates. As shown in Figure 1, all isolates (*n* = 744) were mainly merged into two clusters: cluster I included CC30, CC45, CC59, CC398, and CC121, whereas cluster II mainly comprised CC5, CC1, CC15, CC22, and CC25. The MRSA and OS-MRSA isolates (*n* = 76) were grouped into three clades and one singleton based on the dendrogram (Figure 2). The top nine CCs among all students comprised almost 90% of all isolates; however, the distribution of the top nine CCs among the three educational stages varied, with CC30, CC45, CC5, CC1, C944, and CC7 exhibiting significant differences. The prevalence of CC45 and CC944 isolates was significantly higher in elementary school students; CC30 was significantly higher in junior middle school students; and CC5, CC1, and CC7 were significantly higher in senior middle school students.

### Specific CCs Are Linked to Antibiotic Resistance Patterns

**Figure 3:**
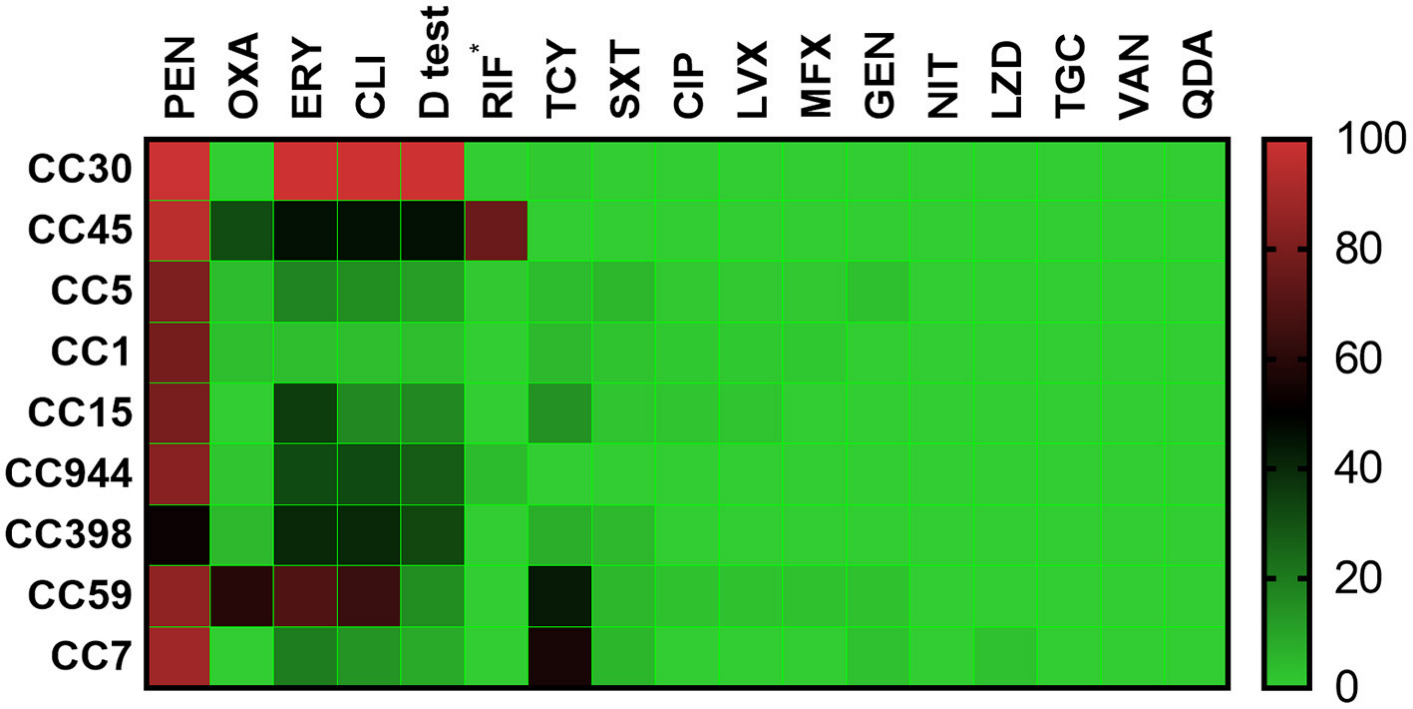
Heatmap of the antibiotic resistance patterns among the top nine clonal complexes. *rifampicin resistance or intermediate resistance. Antibiotics: PEN, penicillin; OXA, oxacillin; ERY, erythromycin; CLI, clindamycin; D test, inducible clindamycin resistance; RIF, rifampicin; TCY, tetracycline; SXT, sulfamethoxazole-trimethoprim; CIP, ciprofloxacin; LVX, levofloxacin; MFX, moxifloxacin; GEN, gentamicin; NIT, nitrofurantoin; LZD, linezolid; TGC, tigecycline; VAN, vancomycin; QDA, quinupristin/dalfopristin.

As shown in Figure 3, all CC30 isolates, as the most prevalent CC, were resistant to ERY and CLI with a positive D test, and most isolates were also resistant to PEN (99.2%). The isolates from CC45, the second most prevalent CC, exhibited very high resistance or intermediate resistance rates to RIF (75.8%), and most were resistant to PEN (95.2%). The isolates from CC1 had the lowest resistance rates to ERY (3.6%) and CLI (3.6%), along with the lowest D test-positive rate (3.6%). The CC7 isolates exhibited very high resistance rates to TCY (55.6%) and showed resistance to LZD (2.8%). The CC45 and CC59 isolates exhibited high resistance rates to OXA at 31.5 and 59.0%, respectively, and most (82.7%) MRSA isolates in this study belonged to these two CCs.

## Discussion

This study targeted school-aged children (*n* = 2,375) from three educational stages (6–18 years) across five schools in Guangzhou, representing a large-scale surveillance study for *S. aureus* nasal colonization and to investigate related antibiotic susceptibility and bacterial genetic structures. The overall prevalence of nasal carriage by *S. aureus* among students at the three educational stages (31.3%, 95% CI: 25.9–36.7%) was much higher compared to those of healthy people, medical college students, and medical laboratory staff in China and worldwide (30–33). The overall prevalence of MRSA (3.0%, 95% CI: 0.6–5.4%) was in the upper range (34), whereas that of OS-MRSA (0.2%, 95% CI: 0.1–0.3%) was relatively low at ~0.5% in cases of *S. aureus* (35). This finding that nasal carriages of *S. aureus*, specifically MRSA, were significantly higher among children at earlier educational stages agreed with several other studies (32, 36).

Previous CLSI guidelines suggested using penicillinase-resistant penicillin, oxacillin, for screening of MRSA isolates, and thus OS-MRSA isolates are prone to be misinterpreted as MSSA (37). In this study, four OS-MRSA isolates were detected. Although the composition was relatively low, careful monitoring is required to detect increases in the prevalence. In 2008, the CLSI recommended using cefoxitin as a surrogate in routine laboratory detection of MRSA (13). Because cefoxitin is a more potent inducer of *mecA*, the cefoxitin screening method was more effective for detecting MRSA. Most OS-MRSA, borderline oxacillin-resistant *S. aureus* could be detected. However, oxacillin/cefoxitin-susceptible *mecA*-positive *S. aureus*, also known as “stealth” MRSA, may still be misinterpreted as MSSA if detection of *mecA* or the Pbp2a protein cannot be performed. In this study, an OS-MRSA strain was found to be susceptible to both cefoxitin and oxacillin phenotypically, namely oxacillin- and cefoxitin-susceptible MRSA, which was not detected by conventional phenotypic susceptibility tests, and thus may lead to therapeutic failure due to misidentification (38). It was recently reported that genetic mutations in the *mecA* promoter region, *gryA/grlA* genes, and *femA* gene were linked to borderline oxacillin-resistant *S. aureus* and OS-MRSA (39, 40). The *mecA* sequence instability and the mutation in the bla regulation system of *mecA* expression were reported to lead to the occurrence of oxacillin- and cefoxitin-susceptible MRSA (15, 16). Based on the amplification and sequencing of the full-length *mecA* and bla regulation system by a commercial supplier (Beijing Genomics Institute), mutations in *mecA* and the bla regulation system of the oxacillin- and cefoxitin-susceptible MRSA in this study were not observed. Thus, other underlying mechanisms require further investigation.

A total of 91 STs was identified among the *S. aureus* isolates, 40.7% of which were novel, suggesting that the nares are important reservoirs for diverse and emerging *S. aureus* genotypes. However, the top nine CCs represented 86.7% of all isolates, suggesting that fluctuation during evolution is mainly limited within the major *S. aureus* lineages. Based on the ST types, the top three MRSA STs were ST45 (45.1%), ST59 (24.0%), and ST338 (5.3%). According to the phylogenetic tree in Figure 2, the MRSA and OS-MRSA isolates (*n* = 76) were grouped into three clades, mainly dominated by CC45, CC59, and CC1. However, CC45 was the most predominant clone (52.0%) among the MRSA isolates and was found at a significantly higher rate in elementary school students. This finding suggests that CC45 has replaced CC59 as the most prevalent MRSA clone among respiratory tract samples from students in southern China (24). In most cases, the prevalent clones among MRSA and MSSA vary in terms of MLST. ST45 and ST59 were two major MRSA clones in China (24); however, in this study, a proportion of these two clones was MSSA. ST45-MSSA was reported in our previous publication and other publication (20, 41), whereas ST59-MSSA was seldomly reported in other studies. In contrast, ST30 was a major MRSA clone in nine Latin American countries (21) but all were MSSA in this study.

CC398 was one of the top nine CCs identified among the present isolates, with two CC398-MRSA (2.7%) isolates being detected. In addition, there were four CC5-MRSA (USA100) and one CC22-MRSA (EMRSA-15) isolates found in this study, which are both well-known hospital-associated MRSA isolates reported in the USA or Europe. These findings suggest the possible influx of hospital-associated and livestock-associated MRSA into the community (42).

CC59 accounted for 75.0% of the OS-MRSA isolates, which agreed with several studies in this region (37). In accordance with previous studies, the antibiotic resistance patterns were strictly associated with some specific *S. aureus* CCs (24, 42). For example, resistance and intermediate resistance to RIF were strictly associated with CC45 isolates, and the ERY, CLI, and TCY resistance pattern was generally linked to CC59 isolates. A new and steady linkage was also observed between CC30 and the resistance patterns of PEN, ERY, CLI, and D test, indicating that the most prevalent CC in this region tended to be resistant to the antibiotics abovementioned.

The limitations of the present study include only using nasal swabs for sample collection; combining this method with throat swabs for MRSA detection may be better. Whole-genome sequencing and further investigation should be performed to analyze the underlying mechanism of the occurrence of oxacillin- and cefoxitin-susceptible MRSA. Finally, we did not apply spa typing in this study because of the large sample size; and combining with SCCmec typing for further classification of MRSA may be better.

In summary, a high prevalence of *S. aureus* and MRSA colonization was observed among school-aged children in Guangzhou, China, with clear age-related patterns concerning the prevalence of these pathogens. Nearly half of the identified STs were novel, indicating that CC45 has replaced CC59 as the most prevalent MRSA clone in southern China. OS-MRSA, particularly emerging oxacillin- and cefoxitin-susceptible MRSA, represent an important issue that warrants further investigation. These findings demonstrate the urgent need to strengthen surveillance programs. Moreover, this study provides updated data that can be used to design enhanced control strategies and improve empirical antimicrobial treatments of potentially subsequent *S. aureus* infections among the school-age population in southern China.

## Data Availability Statement

The datasets presented in this study can be found in online repositories. The names of the repository/repositories and accession number(s) can be found below: https://pubmlst.org/bigsdb?db=pubmlst_saureus_seqdef&page=downloadProfiles&scheme_id=1, pubmlst.org.

## Author Contributions

ZZ designed the study and revised the draft manuscript. BL, XL, FG, and YL performed the experiments. JM, XA, JW, XG, and ZX analyzed the data. BL, ZL, CZ, and SG wrote the manuscript. All authors contributed to the article and approved the submitted version.

## Conflict of Interest

The authors declare that the research was conducted in the absence of any commercial or financial relationships that could be construed as a potential conflict of interest.

## Publisher's Note

All claims expressed in this article are solely those of the authors and do not necessarily represent those of their affiliated organizations, or those of the publisher, the editors and the reviewers. Any product that may be evaluated in this article, or claim that may be made by its manufacturer, is not guaranteed or endorsed by the publisher.

## Abbreviations

CC: clonal complex
CI: confidence interval
CIP: ciprofloxacin
CLI: clindamycin
ERY: erythromycin
GEN: gentamicin
LVX: levofloxacin
LZD: linezolid
MFX: moxifloxacin
MRSA: methicillin-resistant *Staphylococcus aureus*
MSSA: methicillin-susceptible *Staphylococcus aureus*
NIT: nitrofurantoin
OS-MRSA: cefoxitin/oxacillin-susceptible *mecA*-positive *Staphylococcus aureus*
PEN: penicillin
QDA: quinupristin/dalfopristin
RIF: rifampicin
ST: sequence type
SXT: sulfamethoxazole-trimethoprim
TCY: tetracycline
TGC: tigecycline
VAN: vancomycin.
